# Walking a mile in their patients' shoes: empathy and othering in medical students' education

**DOI:** 10.1186/1747-5341-3-10

**Published:** 2008-03-12

**Authors:** Johanna Shapiro

**Affiliations:** 1 Department of Family Medicine, University of California, Irvine – School of Medicine, Irvine, California, USA

## Abstract

One of the major tasks of medical educators is to help maintain and increase trainee empathy for patients. Yet research suggests that during the course of medical training, empathy in medical students and residents decreases. Various exercises and more comprehensive paradigms have been introduced to promote empathy and other humanistic values, but with inadequate success. This paper argues that the potential for medical education to promote empathy is not easy for two reasons: a) Medical students and residents have complex and mostly unresolved emotional responses to the universal human vulnerability to illness, disability, decay, and ultimately death that they must confront in the process of rendering patient care b) Modernist assumptions about the capacity to protect, control, and restore run deep in institutional cultures of mainstream biomedicine and can create barriers to empathic relationships. In the absence of appropriate discourses about how to emotionally manage distressing aspects of the human condition, it is likely that trainees will resort to coping mechanisms that result in distance and detachment. This paper suggests the need for an epistemological paradigm that helps trainees develop a tolerance for imperfection in self and others; and acceptance of shared emotional vulnerability and suffering while simultaneously honoring the existence of difference. Reducing the sense of anxiety and threat that are now reinforced by the dominant medical discourse in the presence of illness will enable trainees to learn to emotionally contain the suffering of their patients and themselves, thus providing a psychologically sound foundation for the development of true empathy.

## Background

When someone is sick, disabled, in pain, hurt, or dying, medicine expects an altruistic impulse from the physician. In other words, the physician must draw closer to the patient, putting the interests of other above those of self, even at some sacrifice to oneself. Scholars have tried to determine what constellation of factors propels certain individuals toward altruistic action [1]. Although the confluence of values, personality, and situation is complex, some researchers have posited an altruism-empathy nexus [2], in which empathy is the underlying motivator and enabling force in altruism. According to this theory, the key ingredient to helping is empathy [3]. Without empathy, social exchange theory, which states benefit must always outweigh cost in any action, takes over [4].

In this sense then, empathy for the patient underlies one of the key professionalism goals of medical education; and as such may be considered a kind of bellwether by which to measure the extent to which the fundamental nature of medical practice is changing. Although Landau [5] contemptuously referred to empathy as "the least" of medicine's professional virtues, in fact, if figuring out how to bridge the inevitable distance between doctor and patient is at the heart of good doctoring [6], then empathy may be "the most" important. The American Association of Medical Colleges has identified the development and enhancement of empathy in medical students as a key goal [7] and the Accreditation Council for Graduate Medical Education lists empathy as a component of professionalism [8]. The value of empathy is cited in specialty training guidelines [9-12], and is mentioned as important by trainees as well [13].

Although the reduction of empathy to its behavioral components [14,15] has received intense criticism [16-19], because it is more easily observably translated into daily clinical practice than the virtue of altruism, it has garnered much more direct emphasis in medical education. Training programs use various exercises and learning activities, such as being admitted to a hospital [20,21], accompanying patients on medical visits [22], participating in empathy enhancing communication workshops [23], making home visits[24], engaging in dramatic enactments [25], writing first person narratives about patients [26] or cadavers [27] and reading medically related literature and poetry as ways of helping medical students acquire empathy for the experiences of people with illnesses and disabilities [28]. On a broader scale, critical contributions to the art of medicine have been made through theoretical and teaching models such as biospychosocial [29], patient-centered [30] and relationship-centered [31] doctoring, all of which have promotion of empathy among learners and practitioners as a key goal. Narrative medicine [6] has recently provided a way of understanding patient-doctor interaction that develops emotional and cognitive skills of narrative competence, enabling appreciation of story as a way of inspiring more empathic and compassionate action on the part of the physician.

Yet, discouragingly, available evidence indicates that empathy tends to decrease among learners during medical school; and even more so during residency training [32-34]; for a contradictory finding, see [35]. As their education progresses, students become cynical and disillusioned [36-38]. Marcus [39] notes that first year medical students relate strongly to patients, while in the third (clinical) year of medical education, they are motivated to counter-identify with patients, and instead are drawn to the doctors whom they have idealized as healthy, invulnerable, authoritative, skilled, and effective individuals who possess powerful and still somewhat mysterious knowledge and skills [40,41].

What is going wrong? We have brilliantly conceptualized models of doctoring that put empathy, in addition to other crucial humanistic qualities, front and center in their doctrines. We have specific behavioral skill-building exercises that are documented to promote empathy. Yet we do not see the dramatic shifts toward greater empathy that we would expect [42,43]. In part, of course, it is appropriate and desirable that trainees become identified with physicians through the socialization process they undergo. It is also true that students need to curb excessive emotional identification with the patient, so that they are able to achieve clinical empathy by comprehending both the patient's perspective and larger, complementary contexts which may be valuable to the patient in coming to terms with his or her medical predicament [44-46]. Nevertheless, medical education still seems surprisingly ineffective in helping students walk a mile in their patients' shoes, as they are so often enjoined to do.

The remainder of this article suggests that true empathy may be more complicated to cultivate toward patients than initially appears and less susceptible to behaviorally-oriented skills training or rhetorical expostulation. We must excavate more deeply to understand what interferes with learners' impulses and desires to express empathy toward patients, especially when available paradigms apparently encourage them to do so. I posit that ingrained ways of thinking symbolically about illness and health, and therefore about what unites and what separates the ill and the non-ill, create barriers that significantly interfere with the development and expression of empathy. I further argue that to truly enhance students' empathy, medical education will require complementary ways of reflecting and teaching that creatively explore and challenge the nebulous and often frightening borderland between patient and physician [47].

## Contamination and othering in relation to health and illness

Despite its pivotal role in medical practice, the impulse to "draw closer," to become engaged and connected with the suffering other, is far from automatic in human nature. In fact, we have an equal, if not stronger, and opposite impulse to draw back, detach, and separate from the contamination of illness [48-50]. This impulse may well be related to fear of our own suffering and death [51], and likely contains an historically important element of self-preservation. If we did not draw back from contagious disease or physical threat, we might easily encourage our own extinction.

### Cultural/philosophical components

Yet this response of withdrawal has a strong cultural/philosophical component as well. Eastern philosophical traditions, such as Buddhism and Taoism [52,53], place emphasis on a fundamental unknowability in the universe, the impermanence of all things including the self, recurring cycles of life and death, thus seeing death as part of life, and the ultimate unity or oneness of self and others. These ancient doctrines, while they do not eliminate the experience suffering or fear of death, mitigate their intensity and balance resistance to death with acceptance and surrender.

By contrast, in the west, the emphasis is on mastery, with the rational mind and intellect viewed as capable of penetrating and solving the mysteries of the world [54]. In the realm of disease, this means that, with the persistent application of logico-scientific investigation, we can vanquish and overcome disease and disability. The Cartesian dualism that characterizes much of western thought defines illness as the opposite of health; and death as the opposite of life. Since health and life are highly desirable, sickness and death become highly undesirable, events to be feared, avoided, or even loathed.

Western cultural/philosophical thought also emphasizes the importance of the individual self, especially as it is distinguished from the other, but as disability [55-57] and feminist [58-60] scholars have pointed out, by and large the valued self is one that is pure, clean, boundaried, and healthy. Thus, on an individual level, each person yearns for a perfectly healthy body immune to fragmentation and corruption [61,62]. Decay, infection, and disease are viewed as potentially engulfing contaminants that represented a fundamental danger to this idealized self [49]. In this way, because ideally desired, health runs the risk of incorporating a moral dimension, and becoming imbued with attributes of goodness.

On the societal level, the desire for a productive, hard-working citizenry also privileges the healthy body as desirable [63]. Illness that is not quickly resolvable is perceived as unruly, out of control, unpredictable, boundary-crossing, and therefore frightening as a social phenomenon that threatens collective stability [48,49,62]. In this view, the role of medicine is to contain and manage the potential chaos of illness from overwhelming the social fabric

However, this healthy and productive self, fantasized by both society and the individual [64] can never be made truly invulnerable, but rather is under constant assault. Disease, deformation, disability are dangerous precisely because they can so easily infiltrate and pollute the previously pure and healthy body. Within this framework, it becomes understandable that the impulse toward altruism, to draw closer, to the suffering other is often overwhelmed by the equal and opposite impulse to withdraw and avoid

### The othering of the sick person

The psycho-structural proposition of the I/Other split formulated by Lacan [65] and other psychoanalytic theorists [66] and social philosophers [67] highlights the human tendency to mark difference as more significant than similarity and to infer something dangerous and threatening from that difference. According to this theory, we tend to define ourselves not only in terms of self, but in terms of other [68]; not only in terms of who we are, but also in reaction to who we are not, or what we cannot allow ourselves to be. In Eriksonian terms, "positive identity" cannot exist without "negative identity" [69]. We are not able to recognize ourselves as pure, healthy, and good unless we have someone whom we can identify as defiled, sick, and "bad." The more the other can be confused with the self, the more urgent becomes this quest for boundary delineation. Projection is a strategy of self-reassurance that "domesticates" our fears of collapse and dissolution. Once located externally, "the fear of our own dissolution is removed. Then it is not we who totter on the brink of collapse, but rather the Other" [70]. All identities that are threatening, and therefore loathsome, to the clean and pure self, become "other."

The binarism of self-other is never value-free, but implies superior-inferior, dominant-subordinate relationships [71]. Thus, the accidental social goal of modern medicine, as opposed to its clearly conceived medical goal of curing disease, is the strict demarcation of sick from well [45]. The more medicalized we become as a society, the more barriers we must erect between the diagnosed person and the presumably healthy person [72]. Protection of the desirable self from being confused with or engulfed by the threatening other occurs through the concept of borders, which establish a self that is fixed and categorical [30,58,62]. To allow permeability in any form, including acknowledgment of shared vulnerability and suffering, is menacing because it leads to a destabilization of the healthy self [57,68].

### Scapegoating

The most extreme form of othering is scapegoating, or the way in which individuals and groups pursue wholeness and reject the frightening or impure elements of themselves (such as vulnerability to illness and death) by projecting them out onto others [73]. The individual and/or group exclude and reject what they fear, and what appears to threaten their wholeness, in this case their goodness and value. As a scapegoat, the defiled person must be symbolically banished, clearly separated from the rest of the group.,. In the case of the sick, order is either re-established through cure and the return of the temporarily exiled to the healthy community; or through symbolic permanent stigmatization and separation to avoid further contamination.

Scapegoating often manifests toward certain ill persons who are "blamed" for their illnesses. In the early days of the AIDS epidemic, this phenomenon was demonstrated not only in the discourse of the general public, but also among many physicians and nurses [74]. Today, it is tacitly accepted in some medical education contexts that residents can mock obese patients, or blame certain categories of drug-abusing, alcohol-addicted, or homeless patients for their medical problems [75]. More broadly, any ill person who cannot recover runs the risk of being defined as deviant [55], essentialized to the restrictive role of patient, thus losing many of his or her formally respected and valued identity attributes [47] and becoming the repository of everything that is feared and to be avoided in life and in medicine. Through various distancing mechanisms, the patient-victim is defined as the outsider, and the insiders are thus bound together [73]. The ill person operates as the stigmatized, scape-goated other [76] whose social role is to symbolically free the privileged, idealized figure of the healthy self from the vagaries and vulnerabilities of embodiment.

## Modern medicine, health, and illness

Thus far, I have considered social phenomena that are supported in part by certain philosophical/culture assumptions and the psychological reactions they tend to produce. I will now turn to the profession of medicine and examine how it both attempts to address and falls short of responding to the societal/cultural milieu in which it is situated. Before proceeding, a caveat is in order.

Having worked as a medical educator for thirty years, I know from first-hand experience as well as the professional literature that the vast majority of students enter medicine motivated by idealism and the desire to help others. I also know that it is the conscious intention of most of the medical educators who are my colleagues within my own institution and around the country to produce graduates who are empathic and caring as well as competent. Further, I am aware that after emotional "peaks" of cynicism and disillusionment in third year and then again during internship, many physicians-in-training find their way to assuming an empathic stance toward their patients. I do not dispute or question any of this; and as a patient I am very glad for it. However, I do maintain that the philosophical structures and assumptions of medicine do not provide adequate guidance in this pursuit; and therefore trainees are often forced to stumble forward under the catch-as-catch-can mentorship of individual physicians who themselves have haphazardly discovered how to draw nearer to their patients. Therefore, my argument below is offered within the context of respect, affection, and esteem for practicing physicians on the front lines of medicine; and the conviction that the educational system can provide them with much better support and direction in cultivating a path toward empathy.

Modernist medical practice grounded in the assumptions of the scientific method addresses the contradictory human impulses of approach and avoidance toward illness and suffering in unique ways. The explicit goal of medicine has always been to prepare its practitioners to draw closer to their patients, with the intention of providing understanding and assistance. But in the modern era, "drawing closer" is mediated by technology: instead of observing and touching the patient directly, scientific advances often substitute technological intimacy for personal closeness. Understanding is translated as diagnosis and prognosis; and assistance becomes treatment and intervention.

It has been noted [77] that science requires a high level of abstraction to successfully promote theory and the testing of theory. Yet such abstraction, while advancing scientific development, encourages a tendency to think about reality from an exclusively abstract perspective, and to overlook the fact that it necessarily omits other aspects of reality that cannot be accommodated by scientific theory. An inadvertent byproduct of this "spirit of abstraction" is that what is not encompassed by or derived from the scientific paradigm is viewed as secondary, subjective, and unreliable. In medicine, such "unimportant" dimensions usually include all the patient's subjective experiences and reactions. In this sense, modern medicine promotes a kind of *scientific *altruism (cf. [78], "cognate professionalism") that still encourages approaching the patient, albeit as an object of interest, rather than a sympathetic subject. The fear and vulnerability underlying withdrawal are addressed by efforts at mastery and control.

In some respects, the modernist discourse that shapes current medical practice challenges the moral meaning of illness. Modern biomedical discourse focuses on disease conceived in terms of pathophysiological mechanisms, not punishment from God, or signs of moral weakness. Working within the modernist paradigm, Susan Sontag famously exposed the damaging effects of the metaphors that attach to stigmatized illnesses such as cancer and AIDS [79,80]. Modern medicine, by reducing illness to its scientific foundation, ostensibly removes moral judgment.

However, the reductionism and objective positivism that underpin medicine are not morally neutral. Its goals of solution, restitution, and restoration both emerge from and reflect western cultural fears of contamination, impurity, and death, Thus, the "cleanly mechanistic view" that science attempts to impose on suffering actually runs the risk of reducing the patient to a disease, an object, a practice that enhances controllability and safety but reduces empathy.

Obviously, infection and contagion have biomedical meanings, and in this regard, modernist contributions in the public health sector such as clean water, waste disposal, hand-washing, use of antiseptic, and even quarantine, have been invaluable in improving population and individual well-being. However, as with much in the modernist paradigm, conclusions that are sensible and useful from a scientific/medical perspective become unconsciously extended to the social sphere with more disturbing results.

The dichotomization of health from illness [81], and the presumption that illness can be eradicated or cured, for example, have produced invaluable breakthroughs in terms of alleviating and ameliorating physical suffering. However, these assumptions have also inadvertently transformed medicine into a vast enterprise to protect the healthy from the ill, to reassure the healthy that they will not become ill; or if they do unfortunately cross over into the kingdom of the sick [79], to ensure that they can be fixed and returned to normalcy. Disease that conforms to a modernist restitution story is more easily acceptable and less frightening. In the Parsonian view of illness [63] productive workers who become ill are allowed a temporary respite from societal obligations while they are restored to their previous good health, and therefore are once again able to assume their productive function (work). Illnesses that do not fit this paradigm become frustrating and frightening because they suggest restitution is not always possible. Because of the increasingly large number of patients with chronic illness in the U.S. [82], more and more individuals find themselves in this liminal state [83]. It is patients in this category who are most at risk from withdrawal and separation by their physicians, especially physicians in training.

## The translation of modernist assumptions into medical education

Despite important curricular reforms and revisions, medicine at its core remains committed to an educational model that is reluctant to relinquish the modernist paradigm. In light of the discussion above, it is not surprising that patients can evoke feelings of fear, anger, disgust, and horror not only in the non-ill, but also in physicians and trainees, although these are rarely acknowledged [84]. Typically, the modernist paradigm urges physicians take refuge from these "unprofessional" reactions in scientific objectivity. Much of the project of medical education is devoted to promoting this safe, boundaried stance in its learners. It promotes the use of a depersonalized language [85], a way of thinking that prioritizes scientific rationalism, and a distanced professional demeanor [40] that enables its adherents to avoid tackling complex emotional issues in self and/or patient that are experienced as unsafe or threatening [86]. Basically, the modernist model of medical education frames the physician in a competent, heroic role in which fear and vulnerability do not play a part. Therefore withdrawal rooted in fear is perceived as unacceptable. Withdrawal based in scientific objectivity, by contrast, is deemed highly professional. Unfortunately, for trainees, it is easy to confuse the two.

Although ways of contemplating the medical experience that consider problematic physician emotions have been introduced in forms such as Balint groups [87], on the whole the dominant medical discourse into which students are socialized lacks a consistent element of reflection and self-awareness. Further, it encourages students to adopt somewhat limited professional roles that emphasize mastery, control, and an aspiration toward perfectability, in the sense of becoming increasingly fearful of making mistakes [88], thus forcing them to reject or deny more flawed aspects of themselves [89]. Suchman [90] discusses the attraction of this discourse to medical practitioners and learners because of its ability to make accurate predictions, achieve desired outcomes, and maintain apparent control of both health and illness.

The modernist solution of transforming patients into objects or tasks [91], rather than as "beings to be known" [92], consciously understood as a way of avoiding unscientific emotional entanglement, unconsciously also underlines and reinforces the "othering" that occurs in response to the deep-seated, culturally transferred fears of contamination discussed above. Once the patient becomes the other, empathy is no longer necessary. Thus the unintended but likely consequence of the modernist discourse's inability to address issues of contamination and othering is a kind of "system arrogance" [91] in which students may see patients not so much as human beings but as projects to be accomplished or puzzles to be solved.

Because students' educational exposure does not include sufficiently thorough preparation to reflect on, be present with, and come to terms with their fear and anxiety about being contaminated by patients' confusion, loss, vulnerability, helplessness, powerlessness, and suffering – and their own – these difficult emotions become objects of dread, to be avoided at all costs. Attempts at empathy in the face of such enculturated psychological pressures tend to exacerbate rather than diminish the student's own anxiety, and raise the likelihood that students' actions will be motivated more by the need to reduce their own discomfort than by the patient's needs [93,94]. In order to forestall contamination from patients in this figurative, emotional sense, students all too often comply with a hidden curriculum [95] that urges them to disavow encounters that disturb their fixed identity and the apparent order and predictability of the medical system. The necessity of boundary maintenance can create profound emotional gaps between healer and sufferer. In this way, patients find themselves under the care of people whose capacity to connect with and care for them has been significantly disturbed [96], and includes "some lasting estrangement."

In a recent issue of *Academic Medicine *devoted to professionalism, several authors blame the hidden curriculum in medical schools for undermining overtly stated goals and values of professionalism [16,97,98]. Basically, these authors argue that medical school faculty and residents often behave in ways that directly contravene professionalism. Although the explanations for this discrepancy are complex, one possible interpretation is a failure of empathy. Putative physician role-models may prioritize efficiency and productivity over patient-centeredness because the systemic paradigm within which they operate does not cultivate empathy toward the patient nor place the patient at the center of care. Rather, the patient has become a means to other ends. This attitude reinforces objectifying and emotional withdrawal from the patient. Many physician role-models, of course, are able to successfully draw near to their patients. The daily practice of physicians is replete with examples of expressing empathy and solidarity with their patients. Nevertheless, in training, despite efforts to develop empathy as a basis for "drawing near" to the patient, this process does not emerge naturally out of the prevailing logico-scientific model, but instead seems like something "tacked on."

## In search of complementary theoretical approaches to promote empathy

In considering the existing modernist biomedical paradigm, it is first of all essential to recognize its extraordinary contributions to the health and well-being of millions of people. Emerging as a product of the belief that people should not suffer, that human intelligence can be used to address and solve problems of physical suffering, biomedicine has been highly successful in achieving its intended goals: cure and amelioration of thousands of specific disease entities and medical conditions. It would be absurd to argue that the biomedical paradigm has become superfluous; and clearly understanding sickness in pathophysiological terms has been and continues to be of incalculable benefit to the human race.

However, the assumptions of the dominant biomedical discourse have had implications not only for biomedical interventions, where they have generally been highly pertinent and useful, but have spilled over into the promulgation of attitudes and values sometimes detrimental to the emotional well-being of the patient and the medical student alike. I have argued that the biomedical discourse has both distracted physicians from empathy, as being secondary and tangential to scientific intervention with patients; and in some cases has led to an unintentional, but powerful, stigmatizing and othering of patients. A paradigm that pursues and idealizes perfectability in the sense of control and mastery of disease and suffering is at once heroic and judgmental. Its enthusiasm for the perfection of health is admirable in many respects; but it can also leave outside its fold those patients who are unable to conform to its dictates. Further, the reductive empiricism of the biomedical model at a *narrative *level leads an emphasis on solution and restitution [83]. An unintended consequence is that the discourse within this paradigm stigmatizes patients who do not meet the restitution story.

Because of these unintended but real consequences, we must enlarge the focus of medical education to include knowledge from other paradigms that are more relevant to the development of empathy and altruism. Previous attempts cited earlier in this paper have not been sufficiently successful because western society in general, and medicine and medical education in particular, continue to privilege the modernist paradigm in a way that lacks nuance and specificity. The point here is that the primacy of biomedical approaches and attitudes is appropriate *in certain spheres; *but we must acknowledge equal and equivalent sources of other knowledge that may be more relevant, useful, and applicable in other spheres, such as determining how to be in relationship with patients. Biomedical knowledge cannot produce empathy, and inadvertently, through its emphasis on reductionism, positivism, and objectivity, it may produce the opposite. Just as we have logico-scientific premises from which the scientific paradigm emerges, so we need a narrative paradigm [99], grounded in certain philosophical premises about the proper relationship between people, to produce empathy and compassion.

This article has argued that, beneficial and insightful as new paradigms have been, to the extent that they continue to be absorbed into the existing modernist paradigm they will be stifled in their ability to reach their full potential, particularly in terms of assisting students in the development and practice of empathy. When attitudes and assumptions strongly embedded in our collective cultural unconscious are triggered, all of us, including students and teachers, will revert to these familiar ways of thinking and reacting. Unless training acknowledges and helps students to reconsider and work with the core boundary issues involving contamination and othering, even the best of alternative/complementary models can be distorted by the power of the modernist discourse to accommodate unnecessarily detached and objectified doctor-patient relationships. We need conceptual and educational approaches that can help students to not be afraid of and indeed to learn from their strong emotional reactions to patients, through "a mutual experience of joining that results in a sensation of wholeness" [100]. Without acknowledging their shared mortality, frailty, and vulnerability, students will not be able to make much sense of truly caring for others [91].

What is needed in medical education are ways of moving students forward so that they are able to become familiar with their fears of contamination, the temptations toward othering, and learn to be emotionally present with their patients. Within a paradigm normalizing and validating this aspect of human life, with time and appropriate role-modeling, students can learn to experience and express profound clinical empathy without feeling at risk themselves.

### An ethics of imperfection

Rather than trying to attach new ways of being in relationship with patients onto the modernist biomedical paradigm, we need to question the comprehensive primacy of the paradigm itself that leads student-physicians to continue to detach and distance themselves from patients. We might start by formulating an ethics of imperfection, a phrase first used by David Morris [101]. Although Morris does not elaborate in detail on this concept, as I understand it, this moral framework would be anchored in acceptance of the limited control we exercise in life and the imperfectability of life itself. This viewpoint suggests that we must learn to accept as well as to resist bodily vulnerability [102]. An ethics of imperfection would likely draw heavily on the insights of philosophers such as Ricoeur [103] whose philosophical theories could provide a foundation from which humane and empathetic behaviors might emerge not just as checklist behaviors, but as deeply felt moral imperatives.

Based on his awareness of the fragility of the human condition, Ricoeur argued that, although irreducible differences will always separate one person from another, ultimately we are inevitably bound up in a quest for mutual recognition and understanding. He recognized that we are all simultaneously capable and vulnerable. This assumption automatically loosens the role boundaries that confine competence to physicians and vulnerability and weakness to patients. Ricoeur further asserted that selfhood and otherness cannot be separated, once we realize that to be able to see oneself as another implies being able to see another as oneself. In this manner, the suffering of others becomes our own suffering. This position poses a challenge to the implications of scientific objectivity, but offers an important psychological position from which empathy for others will naturally and meaningfully emerge

## Practical implications for clinical training of an ethics of imperfection

Research consistently shows that the most important influence on medical student attitudes and learning are positive physician role-models [104]. An ethics of imperfection requires role-models who express vulnerability, share mistakes, incorporate not-knowing; who are aware of and transparent about their emotional reactions to patients and about working the edge between intimacy and detachment; and most importantly, who acknowledge common bonds of humanity with their patients.

In addition to role-modeling, the goals and objectives of medical education are advanced through curriculum. Here what is required is serious focus on issues such as self vs. other; coping with difficult emotions, specifically fear, anxiety, and the desire to detach from death, dying, decay; and the humane and appropriate acknowledgment and management of medical limitations. Various methods exist to achieve these goals, such as reflective practices and incorporation of medical humanities [105-107]. Additionally, the curriculum could incorporate small group discussions with physician role-models to facilitate understanding of these issues; interactions with patients that focus on the patient as teacher [108], not only about the symptoms of the disease, but about the subjective experience of the disease. Finally, the curriculum should incorporate serious study of moral philosophy, not only from the currently still-favored principalist perspective [109] (where the focus is on what should or should not be done to/for the patient); but contemplating the writings of philosophers such as Ricoeur, Buber [110], and Levinas [111] to explore the significant moral implications that accompany the question of how to be in relation with another.

Many of these activities are already in existence, but too often they exist on the fringes of medical education. Rather than being viewed variously as nice fillips (or annoying wastes of time), they should be treated as central to the heart of medicine. Increasingly, we hear calls not only for curricular modifications, but also for institutional transformation and culture change [42,98]. Such proposals recognize that the basic premises of our medical education system need to be enlarged and humanized. It is fundamental change throughout the system of medical education that will help student-physicians learn to authentically face their fears of contamination, vulnerability, and ultimately mortality; learn to stifle their self-protective impulses toward othering or scapegoating of feared patients; and through all of these interior developments, learn to experience and express empathy and altruistically care for their patients.

## Concluding remarks

**Figure 1:**
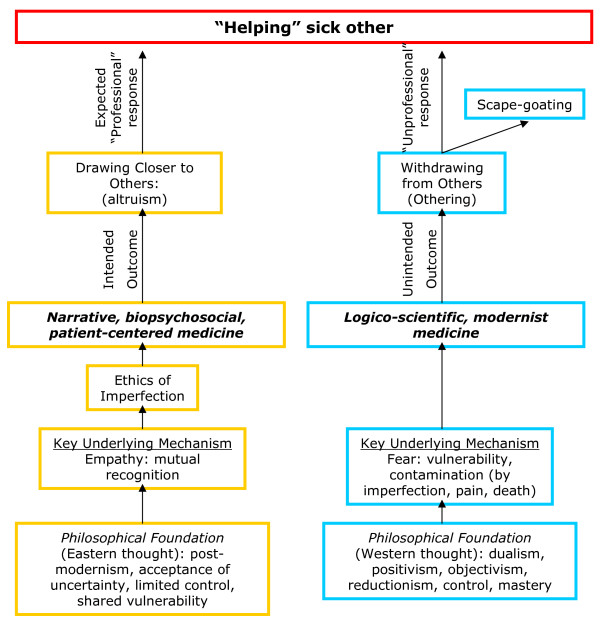
A model for conceptualizing the relationships between empathy and an ethics of imperfection; fear of vulnerability and othering; and the desired outcome of helping the sick other.

The valuing of subjectivity and intimacy [112], the recognition of self in the vulnerable, diseased other, could open the door to a healthy permeability and confusion of boundaries [113] between student-physicians and their patients. From this perspective, the patient becomes no longer solely the doctor's "object," but part of the doctor's self. Such a view does not imply abandonment of scientific and humanistic efforts to alleviate suffering; but it does mean that when that is not possible, or only imperfectly possible, students can learn emotional responses other than uncontrollable anxiety and resultant othering. To learn to accept and see imperfection with tolerance, compassion, and recognition requires the ability to see both patients and self with fresh eyes, without preconceived ideas, with curiosity and caring [114,115]. It involves an emphasis on presence, rather than judgment [116]. An ethics of imperfection could help us recognize and explore, rather than reject and flee from, shared similarities with suffering others, while honoring the inexact and incomplete nature of apprehending their unique experiences [117] (see Figure 1).

Through ways of inclusive, rather than exclusive, thinking about illness, pain, and suffering, students could learn to understand and make sense of others' experiences. Working from this starting point, we might more effectively develop an educational environment that honors shared vulnerability of student-physicians and patients; teaches students to see the other not as the dreaded, rejected parts of self, but as an autonomous, reciprocal presence [118]; and accepts proximity to the patient, in the sense of being able to take a stand with the patient in the face of illness and death [119].

Such a complex perspective on imperfection simultaneously acknowledges parallels and strangeness, understanding and incomprehension between learners and their patients, the mutability and inevitable change in self and other [120]. An ethics of imperfection might facilitate learners' engaging in an excavation of their own and others' tragedies and suffering, rather than turning away in dread, because of the ability to contain and accept at a core level all aspects of life [121]. With a tolerance for and recognition of imperfection in self and others, we could more easily recognize the common bonds of students' and patients' humanity: in any given encounter, it might be easier to help students understand and accept that we are all wounded, all imperfect, and we all share our difference from each other with each other. Being able to emotionally contain with compassion rather than fear the difficult realities of the human condition can form the core for formulating a deep and lasting empathy. To see all humanity as flawed, suffering, and struggling enjoins humility, and cultivates common bonds, and the need to treat the other with kindness out of realization that the gap that separates self and other is not as large as we might think.

Ivan Illich wrote, "medicalized health undermines both our cultural and individual capacity to embrace and respond to pain and suffering" [122]. The self of the student-physician must emerge from philosophical assumptions that allow for the examination and integration of internal qualities that are chaotic, disintegrating, vulnerable, or disturbing; and that help students to recognize rejected others as connected to, rather than walled off from the self. A framework that supports provisional, fluid concepts of identity, openness to resemblances between self and others [123], acknowledgment of imperfection and limitation is needed to help medical students overcome fear and develop a deep and abiding empathy toward their patients.

## About the Author

Dr. Shapiro is professor of family medicine and director of the Program in Medical Humanities & Arts, University of California Irvine, School of Medicine. As a psychologist and medical educator, she had focused her research and scholarship on various aspects of the doctor-patient relationship, including physician interactions with "difficult," stigmatized, and culturally diverse patient populations. She is currently writing a book on medical student poetry. She is feature editor of the *Family Medicine *column, "Literature and the Arts in Medical Education," poetry editor for *Families, Systems, & Health*, and poetry co-editor for the e-magazine *Pulse*.
